# Renewed insights into *Ackermannviridae* phage biology and applications

**DOI:** 10.1038/s44298-024-00046-0

**Published:** 2024-08-21

**Authors:** Anders Nørgaard Sørensen, Lone Brøndsted

**Affiliations:** Department of Veterinary and Animal Sciences, University of Copenhagen, Frederiksberg C, Denmark

**Keywords:** Bacteriophages, Phage biology

## Abstract

The *Ackermannviridae* family was established in 2017, containing phages previously classified within the *Myoviridae* family under the *Viunalikevirus* genus. *Ackermannviridae* phages have been increasingly studied due to their broad range of hosts among *Enterobacteriaceae*, and currently, 174 complete genomes are available on NCBI. Instrumental for their wide host infectivity, *Ackermannviridae* phages display a branched complex of multiple Tail Spike Proteins (TSPs). These TSPs recognize diverse surface polysaccharide receptors, allowing the phages to target strains with distinct lipopolysaccharides or capsular polysaccharides. This review gives an updated overview of the taxonomy and hosts of the expanding *Ackermannviridae* family with significant emphasis on recent advances in structural and computational biology for elucidating TSP diversity, structural domains, and assembly of the branched TSP complex. Furthermore, we explore the potential of engineering *Ackermannviridae* phages and discuss the challenges of using transducing wildtype phages for biocontrol. Finally, this review identifies bottlenecks hindering further advances in understanding *Ackermannviridae* phage biology and applications.

## Introduction

Based on novel principles of phage taxonomy using sequence similarity to determine phage relatedness, the *Ackermannviridae* family of bacteriophages was established in 2017 by the International Committee on Taxonomy of Viruses (ICTV)^1^. Phages belonging to the family of *Ackermannviridae* were previously assigned to the *Myoviridae* family within the *Viunavirus* genus, named after the first phage (Vi01) as described in the latest review in 2012^2^. Since then, the number of phages assigned to the *Ackermannviridae* family has grown and now comprises more than 174 whole genome sequenced phages in National Center for Biotechnology Information (NCBI) (January 25th, 2024). In addition, phages within the family have been proposed extensively for biocontrol due to their broad host ranges. Moreover, *Ackermannviridae* phages are often identified in microbiome datasets, suggesting their widespread presence in the environment and the human and animal gut^3–6^. A morphological hallmark of *Ackermannviridae* phages is the branched complex of multiple tail spike proteins (TSPs) visible on Transmission Electron Micrographs (TEMs) forming star-like structures protruding the baseplate of these tailed phages^7–10^. TSPs are often also called depolymerase (DPO), referring to their depolymerizing activity towards their defined polysaccharide receptor at the bacterial surface for initiating infection. Thus, knowledge of TSP diversity and receptors is essential for developing applications targeting specific bacterial hosts^11–13^. This review provides an updated overview of the taxonomy and biology of the growing *Ackermannviridae* family, and the bacterial hosts targeted by these phages. We outline the virion morphology and describe in detail the structural domains of the TSPs and the assembly of the branched complex consisting of multiple TSPs. We give an overview of hosts infected by *Ackermannviridae* and link this to receptor recognition and TSP diversity. Lastly, we discuss the engineering of *Ackermannviridae* phages, the potential challenges of using wild-type phages within this family for applications, and directions for future research.

### Taxonomy and genomes of the *Ackermannviridae* family

The *Ackermannviridae* family is taxonomically divided into two subfamilies: the *Aglimvirinae* subfamily and the *Civirinae* subfamily. The *Aglimvirinae* subfamily contains two genera, *Limestonevirus* and *Agtrevirus*, whereas the *Civirinae* subfamily contains only the *Kuttervirus* genus. The *Agtrevirus*, *Limestonevirus*, *Kuttervirus*, and *Taipeivirus* genera were established in 2017 and are the most extensively studied genera of the family due to the large number of isolated and characterized phages (Table 1). Recently, additional genera (*Campanilevirus*, *Kujavirus*, *Miltonvirus*, *Nezavisimistyvirus*, *Tedavirus*, and *Vapseptimavirus*) have been included in the family. Still, according to the 2021 ICTV report^14^, each of these recent genera contains only a small number of phages, and only a few publications are available^15–17^.

**Table 1:** *Ackermannviridae* phages and their bacterial hosts

Subfamily	Genus	Phages in NCBI^a^	Bacterial hosts	Reference/Genbank
*Cvivrinae*	*Kuttervirus*	104	*Salmonella* and *E. coli*	^13,89^
*Aglimvirinae*	*Agtrevirus*	8	*Salmonella, E. coli, Shigella* and *Enterobacter*	^34,51,73^
*Aglimvirinae*	*Limestonevirus*	14	*Dickeya* and *Pectobacterium*	^9,54,62^
*Aglimvirinae*	unclassified	9	*Dickeya, Salmonella* and *E. coli*	^27^
	*Campanilevirus*	2	*Vibrio*	MT366762.1MH375644.1
	*Kujavirus*	1	*Vibrio*	MN718199.1
	*Miltonvirus*	5	*Serratia*	^17^
	*Nezavismistyvirus*	2	*Erwinia*	^16^
	*Taipeivirus*	20	*E. coli, Serratia* and *Klebsiella*	^10,58,90^
	*Tedavirus*	1	*Aeromonas*	^15^
	*Vapseptimavirus*	3	*Vibrio*	MH363700.1, MK795384.1, OK428602.1
	*Unclassified*	7	*Acinetobacter, Ralstonia and Vibrio*	2175602

^a^The number of phages infecting each genus was retrieved on January 25th, 2024 at NCBI.

Many *Ackermannviridae* phage genomes have been submitted directly to the NCBI without further characterization. Currently, genomes of *Ackermannviridae* phages deposited at NCBI are between 143 and 164 kilobases in size, with kuttervirus Rabagast carrying the smallest genome and nezavisimistyvirus vB_EamM-Bue1 the largest^18,19^. The genomes share an overall common organization. However, unlike *Tevenviruses*, which organize their genes in functional modules clustering structural, replication, and nucleotide metabolism genes, *Ackermannviridae* phages exhibit a more scattered genomic organization^2,7,20^. Another characteristic of *Ackermannviridae* phages is the hypermodifications of the genomes that were first believed to be hydroxymethyl-uracil substituted instead of thymine^21–23^. Later, a chemical study showed that the genomes of kuttervirus Vi01 and CBA120 were more likely to use a hypermodified thymidine called 5-(2-aminoethoxy) methyluridine (5-NeOmdU) and that the nucleoside substitutes approximately 40% of the thymidine in the genomes^24^. Other studies have demonstrated that all genes necessary for genome modification are highly conserved in the *Ackermannviridae* family, suggesting that all phages undergo this genomic modification^24,25^. While the role of the 5-NeOmdU nucleoside is not fully understood, it has been shown that 60% of commercially available endonucleases fail to cut DNA containing 5-NeOmdU in vitro^26^. Thus, it has been speculated that the modification is a defense mechanism against restriction-modification systems, CRISPR-Cas systems, and other nucleases^25^, indicating that *Ackermannviridae* phages may resist many bacterial defense mechanisms targeting the genome. Furthermore, recent comparative genomics of 14 *Agtrevirus* phages revealed variation in the presence of genes involved in nucleotide metabolism like nicotinamide phosphoribosyl transferase (NAMPT) and ribose-phosphate pyrophosphokinase (RPPK) as well as diversity within genes encoding homing nucleases, along with several other genes that all were annotated as hypothetical proteins^27^. Some phage defense systems deplete the cells of nicotinamide adenine dinucleotide (NAD+), depriving them of essential molecules necessary for replication. Thus, phages carrying genes like NAMPT and RPPK that are part of the production of NAD^+^ could be a way to evade the defense mechanism^28^. While there are diverse genes in the genomes of *Ackermannviridae* phages, the gene cluster encoding tail spike proteins (TSPs) responsible for host recognition exhibited the highest diversity^27^. The TSPs of *Ackermannviridae* phages have been studied in recent years, and their structure, assembly, and diversity are described in further detail below^29–33^.

### Virion morphology of *Ackermannviridae* phages

**Fig. 1:**
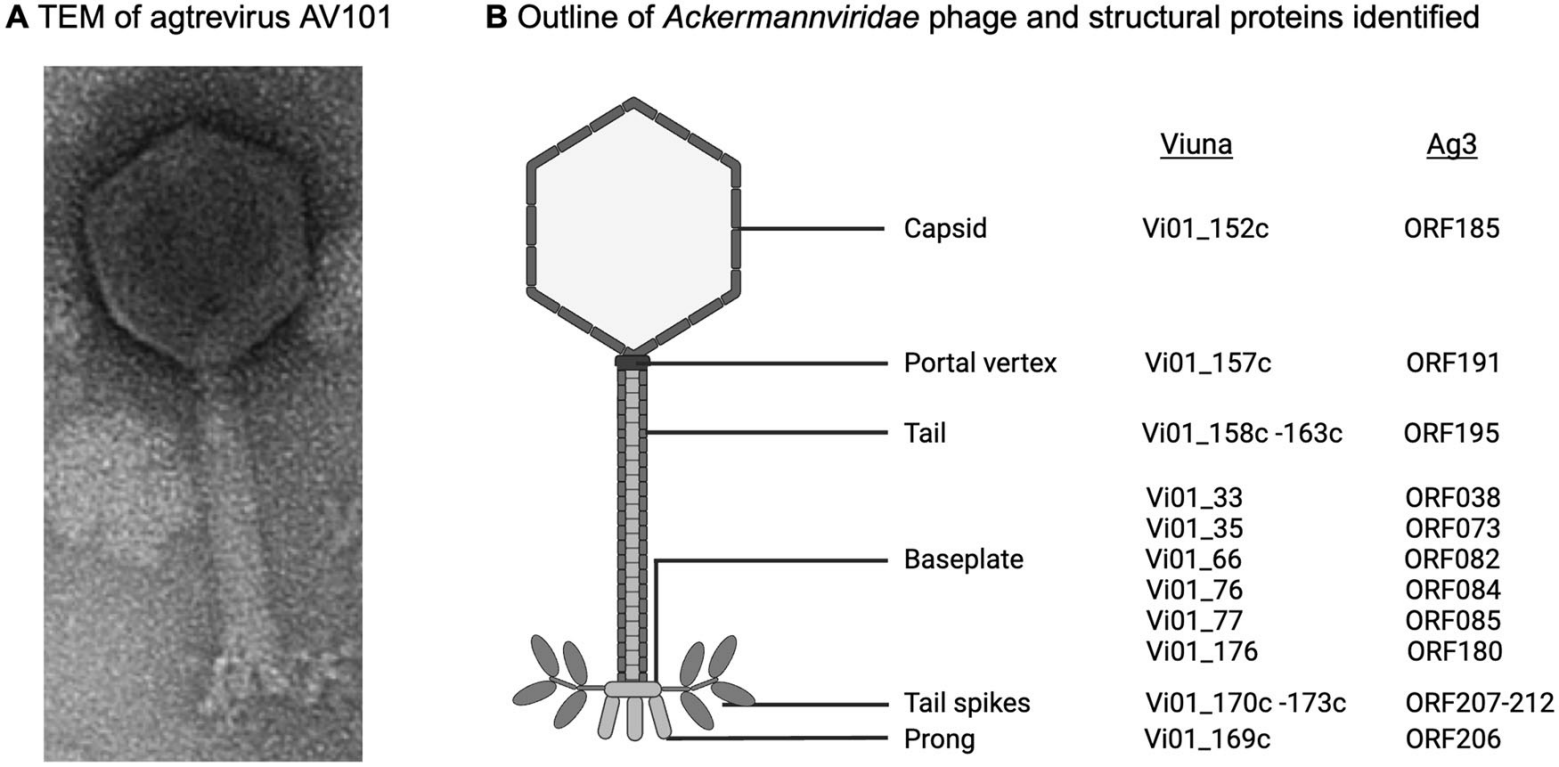
Morphology of *Ackermannviridae* phages. **A** Transmission electron microscopy (TEM) image depicting the morphology of agtrevirus AV101. The icosahedral head is attached to a contractile tail. The tail components include a T4-like neck with a collar, a sheath that envelops a tail tube, and a baseplate with prong-like structures and unique star-like structures at the distal tail. **B** The outline of the morphological characteristics of *Ackermannviridae* phages and their structural proteins determined in kuttervirus ViI and agtrevirus AG3 based on proteomic analysis^7,34^.

Phages within the *Ackermannviridae* family display a contractile tail and head and tail dimensions resembling phage T4, leading to their previous classification as T4-like phages^7,23^. The icosahedral head is around 90 nanometers (nm) wide, while the contractile tail is ~110 × 18 nm. As reviewed in ref. ^2^, the tail components consist of a T4-like neck with a collar, a sheath enclosing a tail tube, a baseplate, and a star-like structure at the distal tail (Fig. 1A, B). Proteomic analysis of kuttervirus ViI and agtrevirus AG3 identified 41 proteins associated with tail morphogenesis, and most of these proteins are homologous to those found in T4 phages^7,34^. However, the baseplate proteins in *Ackermannviridae* phages differ from those in T4, lacking the peripheral part (Gp9-Gp11) responsible for attaching the tail fiber network as well as the small tail fiber (Gp12)^30,35^. Instead, they encoded several other proteins predicted to form the baseplate, yet further experiments are needed to confirm their function (Fig. 1B). Inspecting the TEMs of *Ackermannviridae* phages has revealed prong-like structures protruding from the baseplate, still of unknown function and composition. Yet, it may be comprised of the virulence-associated protein (VriC) annotated in many *Ackermannviridae* phages, as *in-silico* analysis has shown that VriC resembles baseplate proteins encoded by *Listeria* phage A511 and *Staphylococcus aureus* phage phi812^29,36,37^. While the function of these prong-like structures remains to be elucidated, it could be speculated that the protein may aid in host recognition, as it faces downwards of the baseplate, making it likely to encounter the bacterial surface. For example, the prong-like structures may control *Ackermannviridae* DNA ejection, like the tail needle of P22, and thus influence the kinetics of DNA release^38^. While LPS isolated from *S*. Typhimurium or *S*. Anatum hosts was sufficient for DNA release from the capsid of kuttervirus Det7 with a rate of ejection similar to that of lederbergvirus P22^39,40^, the kinetics of DNA injection may be important for a successful infection. Finally, the multiple tail spikes of *Ackermannviridae* phages form a branched protein complex attached to the baseplate. This complex can be observed as star-like structures protruding from the baseplate in TEM (Fig. 1A) and is a distinct morphology only seen in phages belonging to the *Ackermannviridae* family.

### The structure of TSPs and the branched TSP complex of *Ackermannviridae* phages

**Fig. 2:**
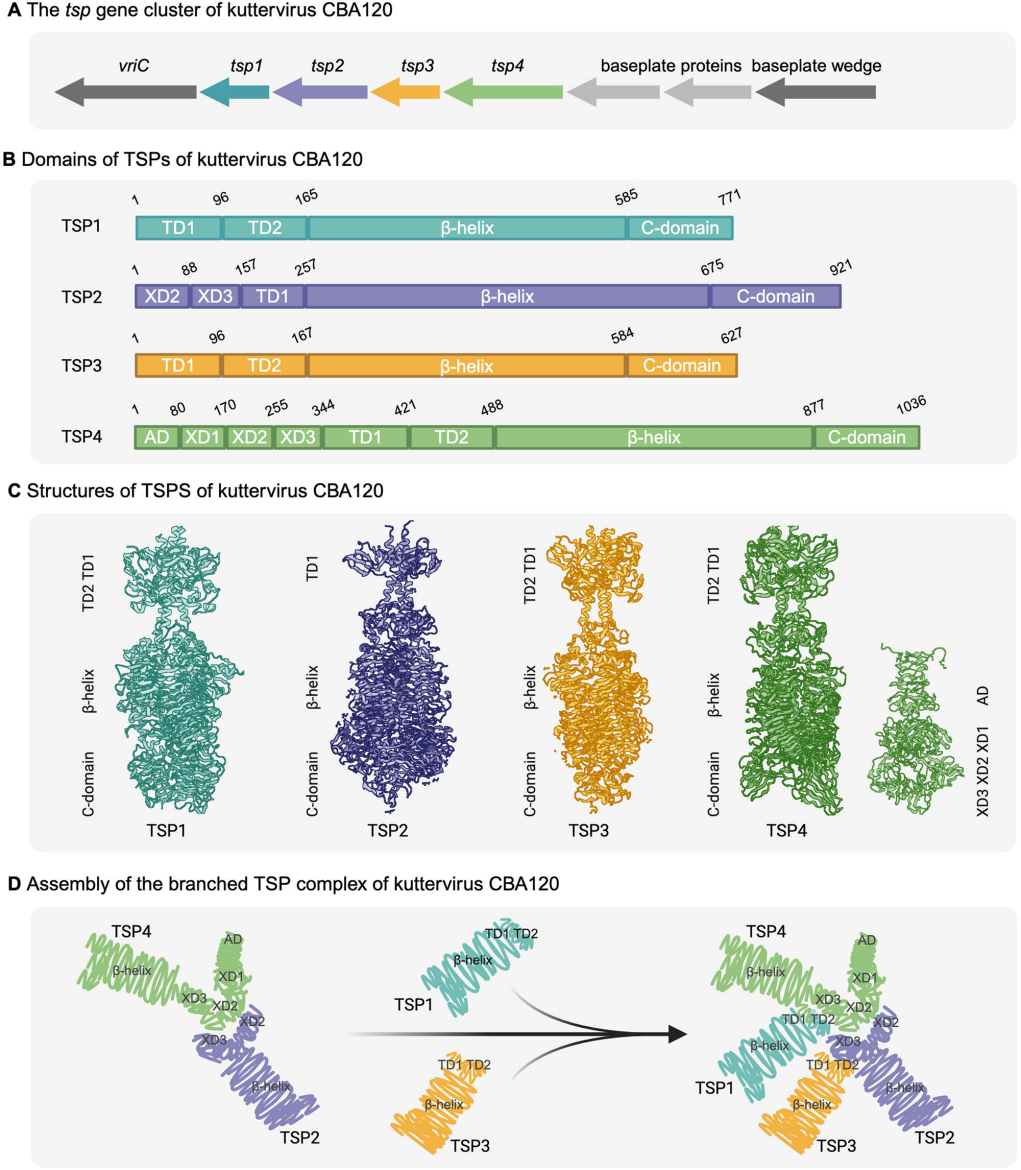
The TSPs of kuttervirus CBA120. **A** The *tsp* gene cluster is a defining genomic trait consistently found in *Kuttervirus*, *Taipeivirus*, *Limestonevirus*, and *Agtrevirus* phages. This cluster is likely prevalent across all *Ackermannviridae* phages. The cluster is located between the *vriC* gene and three baseplate protein-encoding genes. **B** Schematic visualization of the domains found in the four TSPs. All TSPs express a β-helical catalytic domain crucial for host receptor binding. Some TSPs have intramolecular chaperones and carbohydrate-binding domains at the C-terminal end, while the N-terminal region has one or two tandem domains (TD). TSP2 and TSP4 express XD domains that hinge the complex together. The anchor domain of TSP4 (AD) interacts with the virion. **C** Protein structure of the four TSPs of phage CBA120 with indicated domains along the sides. **D** TSP2 and TSP4 interact to form a complex through XD2 domain interactions. Subsequently, TSP1 or TSP3 are added sequentially through interactions with TSP4 and TSP2, respectively. However, recent studies have shown that TSP1 can interact with both TSP4 and the TSP4:TSP2 complex already formed. In contrast, TSP3 can only engage after the TSP4:TSP2 complex has been established. Adapted from refs. ^29,30^.

The *tsp* gene cluster is found in all phages belonging to *Kuttervirus*, *Taipeivirus*, *Limestonevirus*, and *Agtrevirus* and most likely in all other *Ackermannviridae* phages^13^. The *tsp* gene cluster is generally flanked by the *vriC* gene on one side and three genes encoding baseplate proteins, as seen in kuttervirus CBA120 encoding four different TSPs (Fig. 2A)^29^. In general, the structure of TSPs encoded by *Ackermannviridae* phages is similar to other TSPs, including TSPs encoded by distantly related phages^29,41–43^. Using crystallography, protein structures of all four and two TSPs encoded by kuttervirus CBA120 and Det7 have been determined, respectively^29–33,44,45^. In addition, the structural prediction tool AlphaFold2 has been used to predict TSP structures as recently demonstrated for agtrevirus phage AV101^27^. Here, AlphaFold2 structural predictions confirmed conserved protein folds of all TSPs, e.g., similar folding domains as determined by structural analysis of TSPs of CBA120 and Det7^27^.

Plattner et al. and later Chao et al. specifically studied the four TSPs of kuttervirus CBA120 to understand the structure and assembly of the branched TSP complex^29,30^. The structural analysis demonstrated a modular architecture dividing the TSPs into different structural domains. All TSPs are trimeric and contain a large β-helical catalytic domain responsible for receptor binding and degradation of the polysaccharide receptor. The receptor binding capability varies between different TSPs. For instance, the TSP of the phage P22 can bind to the O4 O-antigen as a single monomer (Intra-subunit)^41^. On the other hand, TSP2 of CBA120 O157 binds to the O-antigen between two monomers (Inter-subunit). Upon binding to the receptor O157, the TSP2 of CBA2120 breaks down the polysaccharides into repeating Glc-GalNAc-Rha4NAc-Fuc tetra-saccharide^29^. This receptor degradation is believed to be a common property of all TSPs.

Some *Ackermannviridae* TSPs include an intramolecular chaperone or carbohydrate-binding domain at the far C-terminal part (Fig. 2B, C)^29–31^. In the N-termini of the TSPs, one or two tandem domains (TD) are located. Moreover, TSP2 and TSP4 of CBA120 contain N-terminal XD domains similar to phage T4 Gp10^29^. Gp10 is among the proteins forming the peripheral part of the T4 baseplate responsible for attaching the tail fiber network to the virion and is composed of four different domains^35^. The three first domains of Gp10 are structurally similar to domains found in TSP2 and TSP4 and are referred to as XD1, XD2, and XD3 in *Ackermannviridae* phages (Fig. 2B, C)^13,29,30^. TSP2 contains XD2 and XD3 at the N-terminal associated with the β-helix catalytic domain, whereas TSP4 contains XD1, XD2, and XD3 domains. Furthermore, the distal N-terminus of TSP4 contains an anchor domain (AD) that is believed to interact with the baseplate of the virion (Fig. 2B, C)^29,30^. Gp10-like domains are found in other phages than *Ackermannviridae* and T4 phages. A comprehensive in-silico analysis of *Klebsiella* phages found that the Gp10-like domain could be found in several phage genera, including *Przondovirus*, *Drulisvirus*, and *Alcyoneusvirus*^46^. Like *Ackermannviridae* and T4 phages, the Gp10-like domains function as an adapter or branching protein in phages with multiple receptor binding proteins^46^.

The XD2 and XD3 domains of TSP2 and TSP4 in *Ackermannviridae* phages are crucial for assembling the branched TSP complex. While the XD2 domains of TSP2 and TSP4 interact, their XD3 domain functions as docking stations for the TD domain of TSP3 and TSP1, respectively (Fig. 2D). The TD1 domain of TSP1 in CBA120 is positively charged, allowing for electrostatic interactions with the negatively charged XD3 of TSP4, while the TD1 domain of TSP3 does not have this positive charge. Hence, it still needs to be clarified how TSP3 interacts with TSP2^29^. Plattner et al. further showed that the TSPs were assembled in a specific order to form the branched complex (Fig. 2D)^29^. First, TSP2 and TSP4 form a complex through XD2 domain interactions, followed by the addition of TSP1 or TSP3 through interactions with TSP4 and TS2, respectively. Thus, TSP1 and TSP3 do not seem to depend on binding in any order. However, a recent study showed that TSP1 may interact with TSP4 and the TSP4:TSP2 complex, while TSP3 only interacts once the TSP4:TSP2 complex has formed^30^. Thus, this indicates that TSP1 may interact with TSP4 even before TSP2 is present in the complex, whereas TSP3 does not interact with TSP2 before the complex formation of TSP2 and TSP4. Interestingly, structural predictions suggest that the XD2 domains of TSP2 and TSP4 are conserved in all *Ackermannviridae* phages, while the XD3 domains are not always conserved^13^. Thus, the branched TSP complex assembly may be preserved within the *Ackermannviridae* family’s phages.

### The host range of *Ackermannviridae* phages is influenced by TSP specificity

The *Ackermannviridae* phages are known to infect bacterial species belonging to *Enterobacteriaceae* consisting of gram-negative rod-shaped bacteria, including many that are pathogenic to humans or animals. The hosts of *Ackermannviridae* phages in each genus are shown in Table 1. While *Kuttervirus* phages have been reported to infect *Salmonella, E. coli*, and *Citrobacter*^8,47–50^, *Agtrevirus* phages infect these two species along with *Shigella* and *Enterobacter* species^11,51–53^. Thus, both phage genera infect bacterial species found in the gut of humans and animals, yet *Agtrevirus* seems to have a broader selection of bacterial species as hosts than *Kuttervirus*. In contrast, *Limestonevirus* phages, which belong to the same *Aglimvirinae* subfamily as *Agtrevirus*, infect plant pathogen species such as *Dickeya* and *Pectobacterium*^54–57^. *Taipeivirus* phages can infect *E. coli*, *Serratia*, and *Klebsiella* species^58–60^. Other less studied genera include phages infecting *Vibrio*, *Erwinia*, and *Aeromonas* species, according to NCBI, and demonstrated in three published studies on tedavirus phiA8-29, nezavismistyvirus phiEa2809, and miltonvirus phiMAM1^15–17^. While *Ackermannviridae* phages are initially categorized as infecting the bacterial species from which they were isolated, they may indeed infect multiple bacterial species. For example, kuttervirus CBA120, EP75, and S117 infect *E. coli* and *Salmonella*^12,29,48^.

**Fig. 3:**
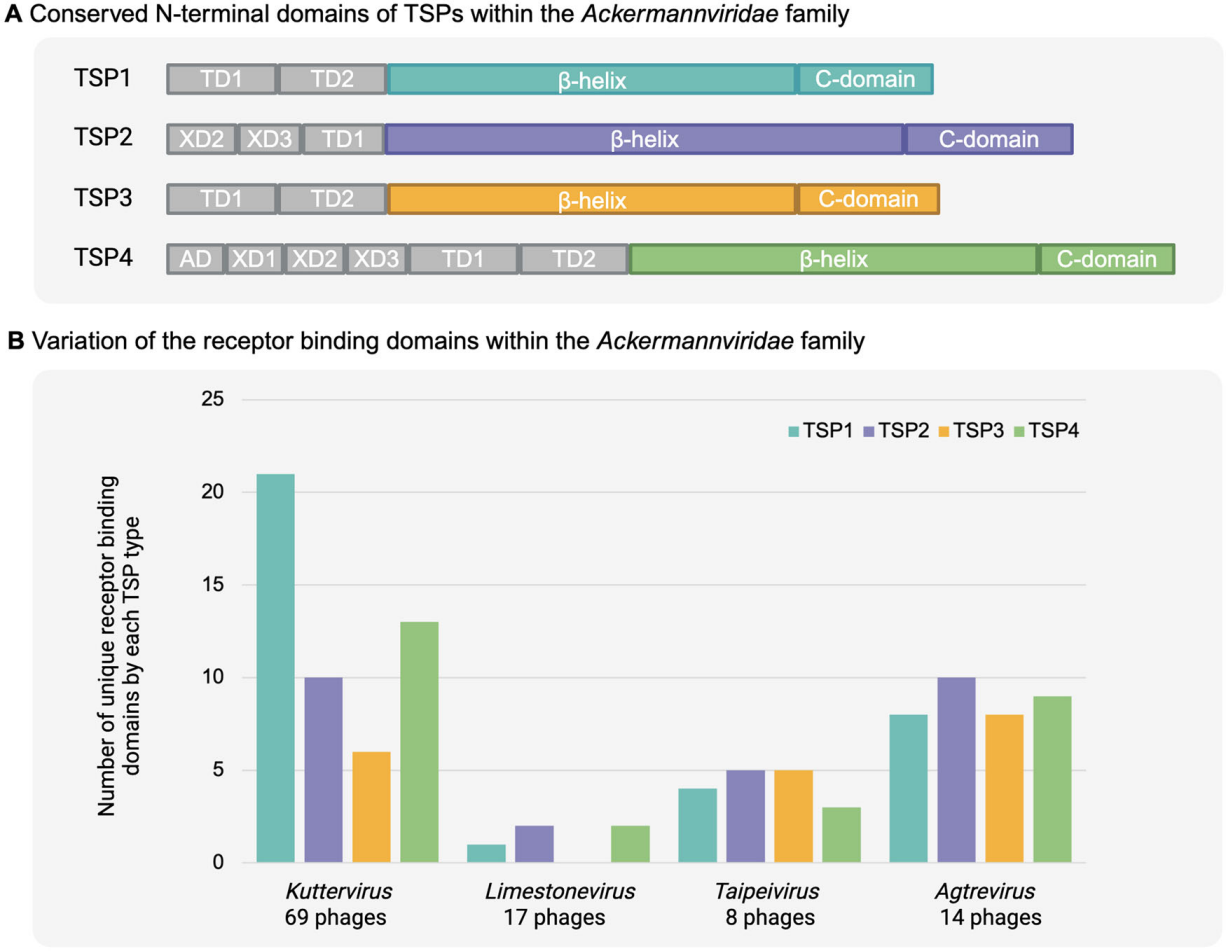
Diversity of TSPs and engineering of *Ackermannviridae* phage engineering. **A** Schematic representation of the conserved nature of the N-terminal of the four TSPs in *Ackermannviridae* phages. In contrast, the C-terminal containing the receptor binding domains is highly diverse. Indication of conserved (gray) and variable (colors) domains in each TSP of *Ackermannviridae* phages. **B** Schematic representation illustrating the extensive diversity of receptor binding domains identified in *Ackermannviridae* phages: *Kuttervirus* (69 phages), *Limestone* (17 phages), and *Taipeivirus* 8 phages) and *Agtrevirus* (14 phages). Adapted from refs. ^13,27^.

Phages of the *Ackermannviridae* family use their TSPs for host recognition. While most *Kuttervirus* and *Agtrevirus* encode four *tsp* genes, some *Taipeivirus* encode up to five^2,13,29,46,61^. In contrast, all *Limestonevirus* analyzed so far only encodes one to three *tsp* genes^2,13,62,63^. Notably, their TSP4 only contains the N-terminal domains, thus lacking the receptor binding domain^13,62^. However, TSPs with receptor binding domains recognize polysaccharides such as lipopolysaccharides (LPS) or capsular polysaccharides as receptors, allowing *Ackermannviridae* phages to infect bacterial strains carrying different polysaccharides at the surface. Such polysaccharides vary substantially within bacterial species, e.g., *E. coli* expresses more than 185 O-antigens of LPS^64^. While the N-terminal of TSPs among *Ackermannviridae* are highly conserved (Fig. 3A), a comprehensive silico analysis of 99 phages in the *Kuttervirus*, *Agtrevirus*, *Limestonevirus*, and *Taipeivirus* genera demonstrated a considerable diversity of the receptor binding domains of the 373 TSPs identified in the study^13^. The TSPs were grouped into subtypes based on the sequence similarity of the C-terminal receptor binding domains (Fig. 3B). For the 69 *Kuttervirus* analyzed, many unique receptor binding domains were identified, including 21 diverse TSP1, 10 TSP2, 6 TSP3, and 13 TSP4. Furthermore, similar receptor binding domains were found in both *tsp1* and *tsp4* genes of kuttervirus phages, indicating recombination as a mechanism for altering host range. For *Limestonevirus*, only 17 phages were available, and they encode only five unique receptor binding domains in their TSP, suggesting less diversity among their TSPs than other genera of the family. In contrast, eight *Taipeivirus* were analyzed and were found to encode TSPs carrying 17 unique receptor binding domains^13^. Later, the analysis of *Agtrevirus* phages was updated and demonstrated that the 14 *Agtrevirus* analyzed encode 35 unique receptor binding domains, thus representing a large diversity within this genus (Fig. 3B)^27^. Overall, the study demonstrated that the receptor binding domains of *Ackermannviridae* TSPs were strongly associated with the phage genera, with a few exceptions, like the receptor binding domain of TSP4 of agtrevirus AV101 is similar to the receptor binding domain of TSP1 of kuttervirus LPST94, suggesting recombination events between phage genera^13^. While receptor binding domains are exchanged between phages in the family, they also share similarities towards receptor binding domains from distant related virulent phages and prophages. For instance, TSP3 of kuttervirus Det7 shares sequence and structure similarity to the temperate lederbergvirus phage P22^44^. Similarly, the receptor binding domain in the TSP1 of kuttervirus CBA120 is similar to a prophage in *Salmonella* Minnesota^29^. Therefore, various mechanisms may allow phages to acquire new receptor binding domains in the *Ackermannviridae* family, emphasizing the complexity of receptor recognition and new hosts of tailed phages.

The ability to recognize multiple diverse O-antigens broadens the range of hosts sensitive to infection by these phages. To understand host recognition of *Ackermannviridae* phages, extensive studies have identified receptors for all four TSPs of kuttervirus phage CBA120 and agtrevirus phage AV101 (Table 2)^27,29^. These results demonstrate that each TSP recognizes specific *E. coli* or *Salmonella* O-antigens. In addition, other studies identified receptors of selected TSPs encoded by several *Ackermannviridae* phages (Table 2). For example, TSP of limestonevirus PP35 recognizes O-polysaccharide consisting of repeating units of 6-deoxy-β-D-altrose of the plant pathogen *Dickeya solani*^54^. In summary, *Ackermannviridae* phages are known to infect a broad range of bacterial species in the *Enterobacteriaceae* family. While receptors for some TSPs have been determined (Table 2), the full extent of their host recognition abilities remains unknown due to the limited number of studies examining their TSPs and host ranges. Notably, such studies could provide datasets for future host range prediction tools and be used to understand the complexity of host recognition of *Ackermannviridae* and other tailed phages. Understanding the host recognition of phages is crucial for using phages in biocontrol, diagnostic, or phage therapy.

**Table 2:** Receptors of *Ackermannviridae* phage TSPs

Phage TSPs	Genus	Host	Receptor	Structure	Accession number	References
**CBA120**	*Kuttervirus*		**LPS**	Crystallography		^29–33^
TSP1 TSP2 TSP3 TSP4		*Salmonella* *E. coli* *E. coli* *E. coli*	O21 O157 O77 O78	4OJ5 5W6P, 6W4Q 5W6F, 6NW9 5W6H, 7RFO	AEM91896.1 AEM91897.1 AEM91898.1 AEM91899.1	
**S117**	*Kuttervirus*		**LPS**			^13^
TSP1 TSP2 TSP3		*Salmonella* *E. coli* *Salmonella*	O21 O157 O4 O9		AXC40875.1 AXC40876.1 AXC40877.1	
**Det7**	*Kuttervirus*		**LPS**	Crystallography		^39^^,44^
TSP2 TSP3		*Salmonella* *Salmonella*	O3,10 O4	2V5I 6F7D	AJQ21022.1 AJQ21021.1	
**EP75**	*Kuttervirus*		**LPS**			^12^
TSP1 TSP2 TSP3		*E. coli* *E. coli* *Salmonella*	O18A O157 O4 O9		AVZ45057.1 AVZ45056.1 AVZ45055.1	
**SPTD1**	*Kuttervirus*		**LPS**			^50^
TSP2 TSP3		*C. sedlakii* *Salmonella*	ND^a^ O4 O9		WBL99284.1 WBL99283.1	
**AV101**	*Agtrevirus*		**LPS**	AlphaFold2		^27^
TSP1 TSP2 TSP3 TSP4		*E. coli* *E. coli* *E. coli* *E. coli*	O8 O82 O153 O159		WJJ54142.1 WJJ54143.1 WJJ54145.1 WJJ54146.1	
**PP35**	*Limestonevirus*		**LPS**			^27^
TSP		*Dickeya solani*	6-deoxy-β-D-altrose		ATW62160.1	
**0507KN2-1**	*Taipeivirus*		**CPS**			^10^
TSP		*Klebsiella*	KN2		BAN78446.1	

^a^*ND* Not determined.

### Engineering of *Ackermannviridae* phages

**Fig. 4:**
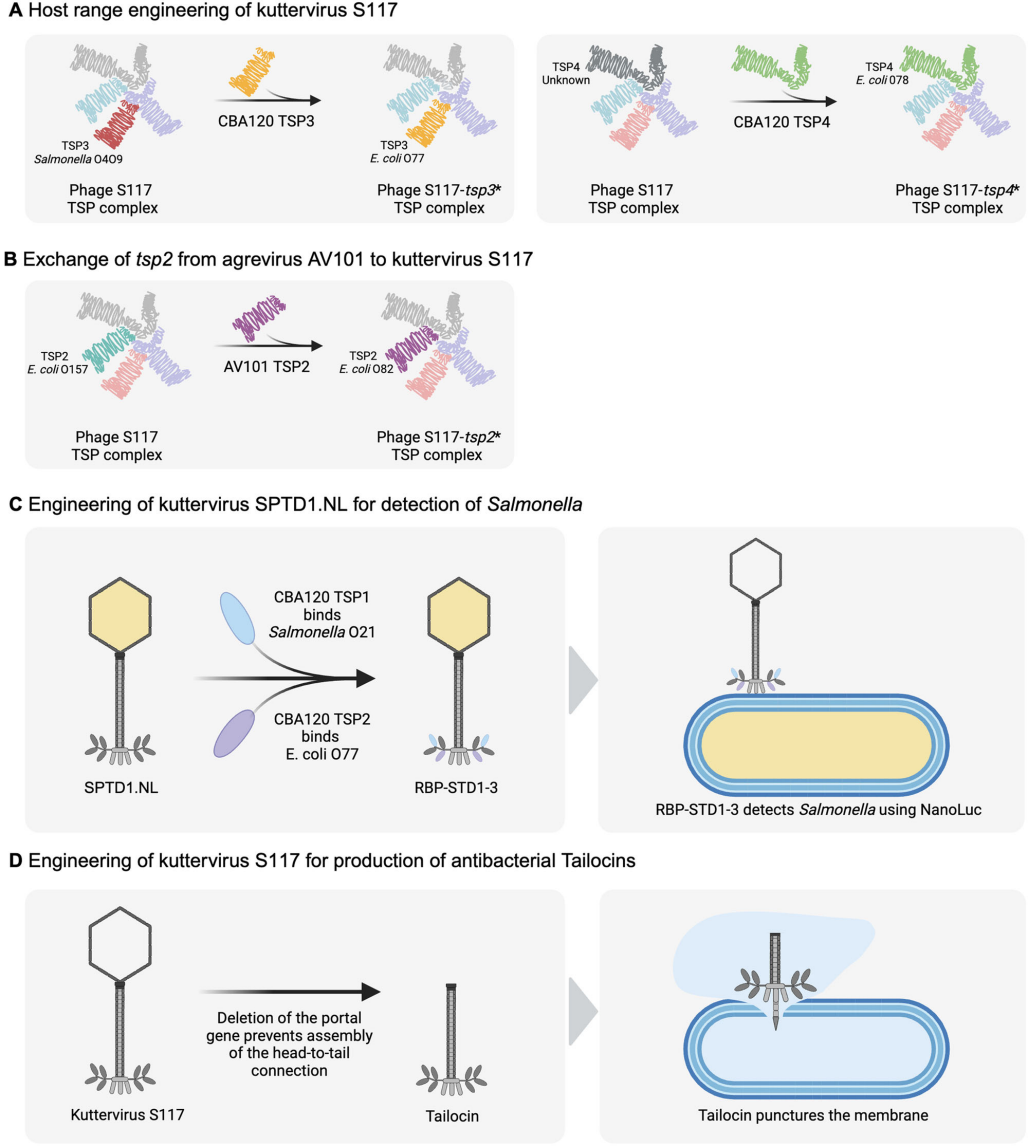
Engineering of *Ackermannviridae* phages and applications of engineered phages. **A** Homologous recombination between kuttervirus S117 and CBA120 allows exchanging *tsp3* and *tsp4* genes, resulting in altered host ranges of the engineered phages S117-tsp3* and S117-tsp4*. **B** Exchange of *tsp2* gene between S117 and agtrevirus AV101 phage resulted in an altered host range for the engineered phage S117-tsp2*. This demonstrates that *tsp* genes originating from different *Ackermannviridae* genera can be exchanged and still produce infectious phages. **C** TSP-engineered STDT.1-NL phage showed superior diagnostic ability for *Salmonella* species compared to the wildtype STDT.1 phage^43^. **D** Genetically engineered *Ackermannviridae* phage S117 created tailocins by deleting the portal vertex or major capsid gene using CRISPR-Cas9. It was shown that tailocin particles from engineered S117 phage could kill the native hosts, *Salmonella* and *E. coli*^69^.

Phage engineering has gained increasing interest in recent years as an approach to optimize phages for applications in biocontrol, therapy, and detection of bacteria. Numerous studies have shown that modifying genes that encode receptor-binding proteins can alter or expand bacterial host recognition by phages^65–68^. Due to their modular structure, *Ackermannviridae* TSPs are excellent targets for engineering receptor recognition due to conserved N-termini domains required for branched TSP complex assembly and variable C-terminal receptor binding domains. Recent studies showed that the *tsp* genes of two *Kuttervirus*, S117 and STDP.1, could be replaced by either entire *tsp* genes or the receptor binding domain from other *Kuttervirus* phages^50^. Both studies used homologous recombination for phage engineering, whereas Sørensen et al. also used CRISPR-Cas9 for counter-selection. Specifically, the *tsp3* and *tsp4* genes of kuttervirus S117 were substituted by *tsp3* and *tsp4* of kuttervirus CBA120, carrying highly similar N-termini but different receptor binding domains. The engineered phages S117-tsp3* and S117-tsp4* now displayed an altered host range according to the exchanged *tsp* genes^69^ (Fig. 4A). Engineering of host range by replacing entire *tsp* genes is not limited to phages within the same genera in the *Ackermannviridae* family, as a conserved N-terminal allowed replacement of the *tsp2* gene of kuttervirus S117 by *tsp2* of agtrevirus AV101^69^. The engineered S117-tsp2* phage could recognize O82 instead of O157 O-antigen on *E. coli*^69^ (Fig. 4B).

The modular structure of the TSPs allows for the exchange of specific domains, such as the receptor binding domain. For instance, the receptor binding domain of *tsp1* and *tsp2* of kuttervirus STDP1.NL was replaced by the receptor binding domain of *tsp1* and *tsp2* originating from kuttervirus CBA120, thereby altering host recognition^50^. In addition, the engineered phage RBP-STDP1-3 could be used as a detection tool for *Salmonella* serovars^50^ (Fig. 4C). To expand host recognition of kuttervirus S117, a fifth TSP was designed to interact with the original TSP complex. Interestingly, the acquisition of *tsp5* resulted in new variants of the branched TSP complex due to the exchange or deletion of other *tsp* genes and did not expand on the host range but still provided a novel TSP, altering host recognition^69^. Another study aimed to produce Tailocins from kuttervirus S117 by genetically deleting the portal vertex gene, creating a headless phage with killing mechanisms similar to pyocins^70–72^ (Fig. 4D). This was previously tried using mechanical methods to remove the head but without success^71^. Instead, the study used different engineering methods like CRISPRi and RNA silencing but only showed that it was possible to create Tailocins by using homologous recombination with CRISPR-Cas9 as counterselection^72^. The engineered Tailocin could kill the different host serovars similar to the S117 wild-type phage. Overall, the studies show that phages in the *Ackermannviridae* family can be engineered to target the hosts of choice but also create novel antimicrobials.

### Application of *Ackermannviridae* phages for biocontrol

The phages in the *Ackermannviridae* family have gained interest in biocontrol or therapeutics because of the broad host range arising from encoding multiple TSPs. Thus, many studies evaluate the potential of *Ackermannviridae* phages in diverse applications^73–81^. For instance, a phage cocktail of kuttervirus EP75 and kuravirus EP335 significantly reduced the presence of *E. coli* O157 on raw beef and vegetables^81^. Another example is the limestonevirus LIMEstone1, which could kill and reduce the number of *Dickeya solani* in a potato tube trail^62^. A recent study tested the effectiveness of a microencapsulated agtrevirus A221 on weaning piglets infected with *E. coli*. The results showed that the piglets who received A221 phages experienced daily weight gain, reduced bacterial load in their tissues and intestinal lesions. The results were comparable to the piglets treated with the antibiotic Florfenicol^82^. Furthermore, kuttervirus phage SPTD1.NL was genetically engineered to express NanoLuc® to detect specific *Salmonella enterica* subspecies^50^. Thus, the phages could be used as a diagnostic tool. However, while it has been demonstrated that *Ackermannviridae* phages can reduce the bacterial population in various biocontrol scenarios, limestonevirus LIMEstone1, kuttervirus ViI and CBA120, and miltonvirus phiMAM1 have all been shown to mediate high general transduction of both plasmids and chromosomal markers with a frequency up to 10^-6^ transductions per PFU ^17^^,^^83^. A similar transduction frequency was also seen in a study that developed Tailocins from kuttervirus S117^72^. Therefore, *Ackermannviridae* phages should not be employed for biocontrol as their potent transduction capabilities may allow transmission of virulence factors or antibiotic-resistance genes to their bacterial hosts. For example, agtrevirus AV101 infecting Extended Spectrum β-lactamase-producing *E. coli* (ESBL)^27^ may potentially spread ESBL plasmids to other phage-susceptible strains. Thus, before using *Ackermannviridae* phages as therapeutic agents, their ability to transduce host and plasmid DNA should be carefully examined.

## Discussion

Phages of the *Ackermannviridae* family exhibit a unique morphology of the distal tail, displaying star-like structures consisting of multiple TSPs and prong-like structures that set them apart from other phages. The diverse host range of *Ackermannviridae* phages covering various bacterial species within the *Enterobacteriaceae* family highlights their potential environmental importance, although more studies are needed to explain their role fully. For example, the transducing abilities of *Ackermannviridae* phages may influence the evolution and fitness of *Enterobacteriaceae* in the gut but has not been investigated so far. While some studies examine the morphology, identify structural proteins, and determine the host range, there remain significant gaps in understanding the full extent of the host recognition abilities of *Ackermannviridae* phages. For instance, the function of the prong-like structure has yet to be determined. In addition, the limited number of studies characterizing specific TSPs and identifying their receptors hinders a comprehensive understanding of the molecular mechanisms underlying host binding and infection. Advanced techniques can enhance our understanding of the prong-like structure, the TSP complex, and their interaction with bacterial hosts. Cryo-electron microscopy and crystallography can present detailed information about the structural features of the TSP complex at near-atomic resolution. Additionally, computational prediction tools such as AlphaFold2 can predict protein structures, making it easier to understand the configuration of the TSPs and the TSP complex. Similarly, the rise of machine learning and prediction tools can, in the future, aid in understanding TSP recognition^84,85^. Already, several studies have used machine learning to predict the tail spike proteins and their hosts like PhageDPO and DePolyermase Predictor^86–88^. By using these advanced techniques, we can gain a better understanding of the precise architecture of the TSP complex and the molecular details that determine its interaction with bacterial hosts. Importantly, investigations of the structural and functional diversity of TSPs and the branched TSP complex among different genera within the *Ackermannviridae* family could provide a more comprehensive insight into their evolution, phage biology, and potential applications.

Phage engineering, as demonstrated by recent studies, holds promise for tailoring the host recognition properties of *Ackermannviridae* phages. *In-silico* prediction of host-phage interactions is improving rapidly, which can be utilized to tailor phage engineering. Moreover, future advancements in protein-ligand interaction prediction will accelerate the engineering of TSP to target specific polysaccharides. This may allow the development of phage-based biocontrol strategies, diagnostics, and therapeutic applications. However, careful consideration must be given to the potential risks associated with the transduction capabilities of *Ackermannviridae* phages, like the dissemination of virulence factors or antibiotic-resistance genes. Therefore, applying these phages in therapeutic contexts should be approached carefully, emphasizing a thorough examination of their transduction capabilities before use. Engineering the specificity of genome packaging may also be a way to create non-transducing Ackermannviridae phages for biocontrol use. Overall, the unique features and untapped potential of *Ackermannviridae* phages deserve further exploration and research in phage biology and biotechnology.
